# Measuring Childhood Disability Using the National Health Interview Survey

**DOI:** 10.1001/jamapediatrics.2025.2910

**Published:** 2025-09-02

**Authors:** Amy J. Houtrow, Chloe S. Shearer, Christina K. Zigler

**Affiliations:** 1 Department of Physical Medicine and Rehabilitation, School of Medicine, University of Pittsburgh, Pittsburgh, Pennsylvania

## Abstract

This survey study examines the prevalence of different strategies for identifying children with disabilities, using questions from the National Health Interview Survey.

In 2019, the National Health Interview Survey (NHIS) adopted the Washington Group on Disability Statistics (WG)/UNICEF Child Functioning Module as the primary strategy to classify children with disabilities.^1,2^ To date, no studies compare the prevalence of childhood disability using the new strategy with other strategies available in the NHIS. This study sought to provide prevalence data for 4 strategies of disability identification, using questions from the 2019 to 2022 NHIS for children aged 5 to 17 years.

## Methods

The NHIS is an annual, nationally representative cross-sectional survey of the noninstitutionalized civilian US population.^2^ One adult member of a household provides information about their own health and the health of others in their family via computer-assisted personal interview (see the eAppendix in Supplement 1 for survey methodology).

This study evaluated 4 strategies to identify disability, including the WG method,^1^ presence of learning/developmental disabilities, receipt of an Individualized Education Program (IEP), and some limitations (having some difficulty with 2 or more core functioning tasks or weekly depression or anxiety) (see the Figure caption for full definitions). The some limitations definition aligns with recommendations from disability researchers and advocates who note that disabilities may be mitigated with assistive technologies and less severe limitations are limitations nonetheless and therefore should be identified.^3^

**Figure.**
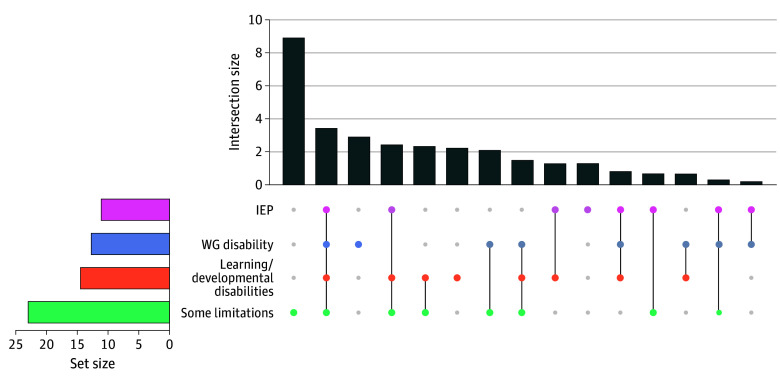
Overlap of 4 Strategies to Identify Children With Disabilities Using the National Health Interview Survey (NHIS), 2019-2022 Each vertical bar shows the mutually exclusive estimated national percentage of children identified by each strategy, indicated by different colored dots and combinations of dots. For example, the first vertical bar represents 9.4% of children identified solely by the some limitations strategy (green dot), while the second vertical bar represents 3.6% of children identified by all 4 strategies (pink, blue, orange, and green dots). Adding all the percentages from each bar provides the total percentage of children identified using at least 1 strategy (33.0%). The 4 disability identification strategies and their survey elements are as follows: (1) WG disability: reported to have “a lot of difficulty” or “cannot do it at all” on 1 or more of the core functioning domains of seeing, hearing, mobility, self-care, communication, learning, remembering, concentrating, accepting change, and controlling behavior or making friends, and/or had “daily” anxiety or depression. (2) Learning/developmental disabilities: presence of current autism spectrum disorder, intellectual disability, learning disability, or attention-deficit/hyperactivity disorder. (3) IEP: reported receipt of an IEP. (4) Some limitations: reported to have “some difficulty” (but not “a lot of difficulty” or “cannot do it at all”) on 2 or more of the core functioning domains of seeing, hearing, mobility, self-care, communication, learning, remembering, concentrating, accepting change, and controlling behavior or making friends, and/or had “weekly” anxiety or depression. IEP indicates Individualized Education Program; WG, Washington Group on Disability Statistics.

Statistical analyses were conducted from February to April 2025, using multiple imputation files and survey weights provided by the NHIS, with missing cases excluded. Prevalence estimates for childhood disability in the general population were calculated using each identification strategy, and an UpSet plot was created in R version 4.4.1 (R Foundation) to show differences in disability identification when using each and multiple strategies.^4^ This secondary analysis follows the AAPOR reporting guidelines, and was considered exempt from review by the University of Pittsburgh Institutional Review Board because the data are publicly available.

## Results

As shown in the Figure, prevalence estimates varied between 11.1% and 23.0%, depending on which disability identification strategy was used. Overlap existed across the strategies such that 7.7% of children aged 5 to 17 years were identified by 2 strategies, 5.4% by 3 strategies, and 3.6% by all 4. An estimated 33.0% of children were identified as disabled by at least 1 of the strategies.

Across all strategies, boys had higher prevalence of disabilities compared with girls, with the difference being most pronounced when identified through receipt of an IEP and select learning/developmental disabilities (Table). Race and ethnicity data were collected because of known disparities in the experience of disability by racial and ethnic group. American Indian and Alaska Native children had the highest prevalence of disability, while Asian children had the lowest. For each strategy, there was an inverse relationship between percentage of the Federal Poverty Level (FPL) and disability prevalence. Using the WG identification strategy, 9.3% (95% CI, 8.8%-9.7%) of children living above 500% of the FPL were identified as disabled, compared with 21.4% (95% CI, 19.5%-22.8%) of children living below 50% of the FPL. The magnitude of difference between less than 50% and more than 500% of the FPL differed by disability identification strategy, from 1.4 times the prevalence using the some limitations strategy to 2.3 times using the WG strategy.

**Table.** Prevalence of Disability by Sociodemographic Characteristics for Children Aged 5 to 17 Years for Each of the 4 Disability Identification Strategies

Characteristic	Prevalence, % (95% CI)			
	WG disability	Learning/developmental disabilities	IEP	Some limitations
Overall prevalence	12.7 (12.0-13.2)	15.5 (14.9-16.2)	11.1 (10.5-11.6)	23.0 (22.1-23.7)
Sex				
Male	13.5 (12.6-14.3)	19.5 (18.6-20.5)	13.9 (13.1-14.7)	24.6 (23.5-25.7)
Female	11.7 (10.8-12.5)	11.3 (10.6-12.1)	8.0 (7.4-8.6)	21.2 (20.2-22.2)
Age, y				
5-11	12.1 (11.2-12.9)	13.7 (12.9-14.6)	10.6 (9.9-11.4)	22.7 (21.7-23.8)
12-17	13.2 (12.4-14.0)	17.5 (16.5-18.4)	11.4 (10.7-12.2)	23.1 (22.1-24.2)
Ethnicity				
Hispanic	11.5 (10.3-12.7)	12.8 (11.7-13.9)	10.5 (9.4-11.5)	21.8 (20.4-23.1)
Non-Hispanic	13.0 (12.3-13.7)	16.4 (15.7-17.2)	11.2 (10.6-11.8)	23.3 (22.4-24.2)
Race				
American Indian or Alaska Native	16.0 (11.5-20.5)	18.4 (14.3-22.6)	17.1 (13.4-20.8)	33.1 (27.7-38.6)
Asian	6.5 (4.9-8.1)	5.1 (3.8-6.5)	5.6 (4.1-7.1)	12.6 (10.5-14.6)
Black or African American	13.5 (11.5-15.4)	15.9 (14.1-17.8)	12.0 (10.4-13.6)	20.8 (18.7-22.8)
White	13.0 (12.3-13.7)	16.8 (16.0-17.6)	11.3 (10.7-12.0)	23.9 (22.9-24.8)
Other^a^	13.6 (11.0-16.2)	15.9 (13.0-18.9)	11.0 (8.5-13.4)	26.3 (23.0-29.6)
Family income, percentage of FPL				
<50	21.4 (19.5-22.8)	21.6 (19.8-22.8)	14.9 (13.1-16.2)	28.2 (27.1-29.0)
50-99	18.1 (17.0-19.1)	20.4 (19.6-21.1)	15.3 (14.4-16.1)	29.1 (28.5-29.7)
100-199	14.1 13.3-14.7)	17.1 (16.5-17.5)	12.6 (11.9-13.1)	24.8 (24.2-25.4)
200-299	11.1 (10.4-11.8)	14.5 (13.8-15.1)	10.5 (9.8-11.2)	21.8 (21.2-22.4)
300-399	10.7 (9.8-11.4)	13.6 (12.9-14.1)	9.1 (8.4-9.8)	20.6 (19.9-21.2)
400-499	11.6 (10.3-12.8)	14.5 (13.6-15.3)	9.5 (8.6-10.3)	20.8 (19.7-21.6)
≥500	9.3 (8.8-9.7)	13.0 (12.6-13.4)	8.7 (8.3-9.1)	19.9 (19.6-20.2)

Abbreviations: FPL, Federal Poverty Level; IEP, Individualized Education Program; WG, Washington Group on Disability Statistics.

^a^ Other single or multiple races.

## Discussion

Similar to other research evaluating childhood disability prevalence,^5^ prevalence estimates varied based on the identification strategy used. Limited overlap between strategies suggests the need for caution when interpreting research based on a single identification approach. When seeking a broader classification of children with disabilities, using more than 1 identification strategy could be advantageous. Because of the potential impacts on needs assessments and resource allocation, critically appraising the method of disability identification is warranted, especially when seeking to identify disability among specific marginalized groups for whom targeted interventions may be needed.^6^ This study is limited by recall bias because the data rely entirely on parent or guardian report, and the presence of a disability was not verified in a medical, therapeutic, or educational setting.

## Notes

Supplement 1. eAppendix

Supplement 2. Data Sharing Statement
