# Supplementary material for: Measuring Childhood Disability Using the National Health Interview Survey
Source: JAMA Pediatr. 2025 Sep 2;179(11):1233–6. doi: 10.1001/jamapediatrics.2025.2910 (PMC12406140; doi:10.1001/jamapediatrics.2025.2910)
Supplement: Supplement 1. — eAppendix [file jamapediatr-e252910-s001.pdf]

# Supplemental Online Content

Houtrow AJ, Shearer CS, Zigler C. Measuring childhood disability using the National Health Interview Survey. *JAMA Pediatr.* Published online September 2, 2025.
doi:10.1001/jamapediatrics.2025.2910

**eAppendix**

This supplemental material has been provided by the authors to give readers additional information about their work.

© 2025 Houtrow AJ et al. *JAMA Pediatrics.*

## eAppendix

This study focused specifically on data provided by a parent or guardian on 1 randomly selected child within their household. The sample child response rate for each year was calculated by dividing the number of parent or guardian–completed interviews for sample children by the number of eligible sample children from households with completed rosters and then multiplying this by the household roster completion rate. The response rate in 2019 was 59.1%, 2020 was 47.8%, 2021 was 49.9%, and 2022 was 45.8%.[1-4] A sample weight variable is included in each year's datafile, calculated to account for the probability of nonselection of minority groups, as well as nonresponse bias among selected households.[5,6] In the pooled 2019-2022 dataset, the weights were adjusted by dividing by 4. The multiple imputation files were provided by the National Center for Health Statistics to address missingness of family income data in the datasets.[7] Family income was measured using the percentage of the Federal Poverty Level, which relies on the US Census Bureau's poverty thresholds.[8]


1. National Center for Health Statistics. 2019 Survey Description National Health Interview Survey *https://ftpcdcgov/pub/health_Statistics/NCHs/Dataset_Documentation/NHIS/2019/srvydesc-508pdf*. 2020 Accessed 2/7/25
2. National Center for Health Statistics. 2020 Survey Description National Health Interview Survey *https://ftpcdcgov/pub/health_statistics/nchs/dataset_documentation/NHIS/2020/srvydesc-508pdf*. 2021;accessed 2/7/25
3. National Center for Health Statistics. 2021 Survey Description National Health Interview Survey *https://ftpcdcgov/pub/Health_Statistics/NCHS/Dataset_Documentation/NHIS/2021/srvydesc-508pdf*. 2022;accessed 2/7/25
4. National Center for Health Statistics. 2022 Survey Description National Health Interview Survey *https://ftpcdcgov/pub/health_Statistics/nchs/Dataset_Documentation/NHIS/2022/srvydesc-508pdf*. 2023;accessed 2/7/25
5. Moriarity C, Parsons VL, Jonas K, Schar BG, Bose J, Bramlett MD. Sample design and estimation structures for the National Health Interview Survey, 2016–2025. *Centers for Disease Control and Prevention*. 2022;accessed 2.25.25(https://stacks.cdc.gov/view/cdc/115394)
6. Bramlett MD, Dahlhamer, J.M., Bose, J., Blumberg, S.J. . New Procedures for Nonresponse Adjustments to the 2019 National Health Interview Survey Sampling Weights. *Division of Health Interview Statistics*. 2020;National Center for Health Statistics(https://ftp.cdc.gov/pub/Health_Statistics/NCHS/Dataset_Documentation/NHIS/2019/nonresponse-report-508.pdf)(Hyattsville, Maryland):accessed June 14, 2025.


© 2025 Houtrow AJ et al. *JAMA Pediatrics.*

7. Zablotsky B, Lessem SE, Gindi RM, Maitland AK, Dahlhamer JM, Blumberg SJ. Overview of the 2019 National Health Interview Survey Questionnaire Redesign. *American Journal of Public Health*. 2023;113(4):408-415. doi:10.2105/ajph.2022.307197
8. National Center for Health Statistics, Division of Analysis and Epidemiology. Poverty. *National Health Interview Survey*. 2024;https://www.cdc.gov/nchs/hus/sources-definitions/poverty.htm(accessed June 15, 2025)

© 2025 Houtrow AJ et al. *JAMA Pediatrics*.
