# Supplementary material for: Measuring Childhood Disability Using the National Health Interview Survey
Source: JAMA Pediatr. 2025 Sep 2;179(11):1233–6. doi: 10.1001/jamapediatrics.2025.2910 (PMC12406140; doi:10.1001/jamapediatrics.2025.2910)
Supplement: Supplement 2. — Data Sharing Statement [file jamapediatr-e252910-s002.pdf]

Data Sharing Statement

Houtrow. Measuring Childhood Disability Using the National Health Interview Survey. *JAMA Pediatr*. Published September 02, 2025. doi:10.1001/jamapediatrics.2025.2910

Data
Data available: Yes

How to access data: https://www.cdc.gov/nchs/nhis/documentation/index.html
